# Interdisciplinary Approaches to Assessing the Health of People Living in Environmentally Adverse Conditions

**Published:** 2019-09

**Authors:** Yerbol BEKMUKHAMBETOV, Arstan MAMYRBAYEV, Timur JARKENOV, Talgar ABILOV, Gulnar SULTANOVA, Gulnar ISAEVA, Mukhtar ZHAILYBAYEV, Raisa URAZ

**Affiliations:** 1. Administration, West Kazakhstan Marat Ospanov State Medical University, Aktobe, Kazakhstan; 2. Department of Hygiene and Occupational Diseases, West Kazakhstan Marat Ospanov State Medical University, Aktobe, Kazakhstan; 3. Department of Molecular Biology and Medical Genetics, West Kazakhstan Marat Ospanov State Medical University, Aktobe, Kazakhstan; 4. Department of Children and Surgical Dentistry, West Kazakhstan Marat Ospanov State Medical University, Aktobe, Kazakhstan; 5. Department of Clinical Anatomy with Operational Surgery, West Kazakhstan Marat Ospanov State Medical University, Aktobe, Kazakhstan; 6. Department of Therapeutic and Prosthetic Dentistry, West Kazakhstan Marat Ospanov State Medical University, Aktobe, Kazakhstan

**Keywords:** Environmental problems, Nonmedical determinants, Child health, Adult health, Environmental risk

## Abstract

**Background:**

The health status of the population of different ages was examined. Since children are very vulnerable to environmental factors, our goal was to examine their health status and compare them with those of the older population. Also one of the important tasks of our study was the installation of carcinogenic and non-carcinogenic risks for children and adults of different sexes.

**Methods:**

During our research, we calculated the air pollution index, investigated the incidence statistics of the population, and calculated the lifetime average daily dose (LADD). We investigated the content of sulfur dioxide, hydrogen sulfide, nitrogen oxide, ammonium and hydrogen carbonates and compared it with safe level of exposure.

**Results:**

In Aktobe, the Republic of Kazakhstan the external environment is polluted with boron and chromium, and in Aktau - with organic hydrocarbons. High morbidity rates in adolescents were found for endocrine disorders, digestive system diseases, and musculoskeletal system disorders. Estimating the prevalence and incidence of newly diagnosed diseases among women in Aktau showed that the overall incidence rate, as well as the incidence of respiratory and skin diseases, declines with age. The incidence of the genitourinary system and the number of nervous disorders increase, and the number of neoplasms increases in men population. A study of carcinogenic risks showed that children with Aktau, especially boys, have the greatest risk of cancer.

**Conclusion:**

The conducted research shows that environmental factors have a big impact on the health of the population.

## Introduction

The increasing man-induced environmental pollution has a pronounced effect on the development of public health care; this problem becomes increasingly relevant with each passing year (1–3). Medical aspects of environmental protection, disease prevention, and strengthening of public health are a crucial world problem (4, 5).

Children are extremely susceptible to the effect of adverse environmental conditions (6–8), since their endocrine, immunocompetent, and histomorphologic systems are underdeveloped, which often causes various diseases. Besides environmental problems, the modern industrial society also exposes children to heavy study loads, accompanied by chronic stress factors, sedentary lifestyle, unbalanced diet, and behavioral stereotypes that put children’s health at risk (9–13).

The evaluation of the link between the qualitative composition of the environment and the morbidity rate, its structure, physical development and puberty indexes, and the rate of endocrine disorders is important for the protection of public health. With that, this system prioritizes the assessment not only of environmental risks, but also of the nutritional status and the characterization of nonmedical determinants.

A breakthrough in any field of scientific research is related to the development of new technologies and methodological approaches. In this aspect, the interdisciplinary approach plays the main role. This approach is the highest form of integrative activity in science. Studies at the intersection of medical disciplines – hygiene, pediatrics, endocrinology, and urology produced interesting scientific material, which is presented below.

Health care issues are important for each country, so this study will help to highlight the health situation in the present tense. It is important to identify the main types of diseases and the risks of their development in order to reduce the number of patients in the future. The purpose of this research was to study the effect of atmospheric pollution on the morbidity rate and biomedical indexes of public health.

## Methods

### Study design

Data on the qualitative and quantitative state of environmental objects (air) for the period from 2005 to 2013 in Aktobe and Aktau (The Republic of Kazakhstan) were obtained (14). The monitoring of atmospheric pollution was based on the information provided by the Committee on Consumer Protection and the administrative statistical report F.18.

The air pollution index (API_5_) (15) was calculated based on five chemical substances with the highest specific maximum permissible concentration (MPC) value, with regard to their hazard category. API is the complex air pollution index that includes several pollutants. The API value is calculated from the average annual concentrations of chemical substances. Therefore, this index characterizes the level of chronic and long-term air pollution. API includes not only the n concentrations of various substances, but also their adverse health effect. The index is calculated as follows:

In=∑=∑(xi/MPCi)Ci,

where Xi is the average annual concentration of substance i; Ci is the coefficient that enables raising the level of air pollution with substance i to the power of air pollution with sulfur dioxide or any other chemical substance; In is the API, a dimensionless quantity. Four categories of air quality were distinguished based on the level of pollution. The level of pollution was considered low if the API was less than five, increased if the API ranged from five to eight, Ci<5, high if the API ranged from eight to 13 and Ci ranged from 5 to 10, and extreme of the API was more than 13 and the Ci was more than 10.

Information regarding the morbidity rate during the last ten years (2004–2013) was gathered from the main departmental medical records on registration and reports (annual departmental statistical report on the morbidity rate – F.12; F.025U, F.026U), and subsequently processed. Information was also extracted from the materials of the Committee of Statistics (16). The indexes of newly diagnosed disease morbidity, its structure and prevalence were used to assess the dynamic of the diseases. The disease prevalence was analyzed according to the WHO 10^th^ revision of the International Statistical Classification of Diseases and Related Health Problems (3). This study was conducted according to the principles of the Declaration of Helsinki and was approved by the Ethics Committee. All the patients provided informed consent prior to enrollment.

### Intervention

The non-carcinogenic and carcinogenic risk to the health of children and adolescents of Aktau was assessed. We investigated the content of sulfur dioxide, hydrogen sulfide, nitrogen oxide, ammonium and hydrogen carbonates (17).

The development of non-carcinogenic effects in Aktau was characterized by comparing the actual levels of chemicals (sulfur dioxide, hydrogen sulfide, carbon monoxide, nitrogen oxides, ammonia, and total hydrocarbons) in the atmosphere with the safe level of exposure (index / hazard ratio) according to the following formula: HQ = AC / RfC, where the AC is the actual level of exposure; RfC is the safe level of exposure.

Based on results of HQ (18), Hi calculation and the recommended standard values of exposure factors, the population risk for adults (men, women) and adolescents was assessed. The calculation of the individual cancer risk was performed using data on the exposure level and the values of carcinogenic potential factors (slope factor, unitary risk). In general, the additional probability of carcinogens causing cancer in an individual throughout life (CR) is estimated, based on the lifetime average daily dose (LADD). The following stage involved the calculation of the lifetime average daily dose (LADD) out of one or several chronic average daily doses (ADDch), as a weighted average dose for three life periods with the following formula (19):

$$L\text{ADD}=\frac{\left(\text{EDb}\times \text{ADDchb}\right)+\left(\text{EDc}\times \text{ADDchc}\right)+\left(\text{EDa}\times \text{ADDcha}\right)}{\text{AT}}$$

where

LADD is the lifetime average daily dose; EDb is the exposure duration of junior children (aged 0–6) – 6 years; EDc is the exposure duration of senior children (aged 6–18) – 12 years; EDa is the exposure duration of adults (aged 18 and above) – 12 years; ADDchb is the chronic average daily dose of junior children, mg/(kg*day); ADDchc is the chronic average daily dose of senior children, mg/(kg*day); ADDcha is the chronic average daily dose of adults, mg/(kg*day);AT is the averaging time (number of years).

### Statistical analysis

Statistical treatment was carried out with SAS software, version 9.2(USA). Normal distribution was assessed by the Kolmogorov-Smirnov test. The results of the study are presented as mean value (M) and standard deviation (SD). A two-sample t-test with different dispersions (t) and the Wilcoxon test (z) were used to evaluate the statistical significance of differences. Correlation analysis was performed by calculating the Spearman correlation paired rho. Criteria values of r<0.05 were considered statistically significant.

## Results

We analyzed the 2004–2013 research data on air pollution in Western Kazakhstan. We would like to note that the most polluted was Aktobe (Table 1). For Aktobe, pollution with chrome and boron was predominantly noted, and in Aktau - with organic hydrocarbons. Analysis of the average incidence rates and newly detected diseases (Table 2) among residents of Aktau in 2004–2013. Showed that in general, the overall incidence rate declines with age (exception: neoplasms, eye and blood system diseases, genitourinary tract). High morbidity rates in adolescents were found for endocrine disorders, digestive system diseases, and musculoskeletal system disorders. Estimating the prevalence and incidence of newly diagnosed diseases among women in Aktau showed that the overall incidence rate, as well as the incidence of respiratory and skin diseases, declines with age.

**Table 1:** The level of air pollution in regional centers

***Cities***	***Air pollution index (API_5_)***									
	***Years***									
	***2004***	***2005***	***2006***	***2007***	***2008***	***2009***	***2010***	***2011***	***2012***	***2013***
Aktobe	9.6	10.1	9.7	9.5	8.5	8.6	7.6	6.9	6.4	4.2
Aktau	4.4	4.0	3.5	4.3	4.5	3.5	3.0	2.6	3.0	3.7

**Table 2:** Characteristics of the prevalence and morbidity rate of newly diagnosed diseases, depending on the age of residents of Aktau (cases per 100000 persons, M±m)

***No.***	***Disease class***	***Prevalence***					***Morbidity rate of newly diagnosed diseases***	
		***Children***	***Adolescents***	***Adults***	***Children***	***Adolescents***	***Adults***
	Number of persons	46116	10111	101802	46116	10111	101802
	General morbidity rate	137740.7 ± 546.5	87769.6 ± 931.7	81892.9 ± 283.6	112741.8 ± 494.4	66523.4 ± 811.1	53425.8 ± 229.1
I	Infections and parasitic diseases	6131 ± 115.3	1645.2 ± 127.5	3671.1 ± 60.0	5796.8 ± 112.1	1096.9 ± 104.1	2322.4 ± 47.8
II	Neoplasms	273.8 ± 24.4	138.3 ± 37.0	859.3 ± 29.0	219.3 ± 21.8	92.1 ± 30.2	587.2 ± 24.0
III	Diseases of the blood, blood-forming, immune system	10939.8 ± 154.0	6708.3 ± 257.6	1592.9 ± 39.5	5118 ± 105.3	4556.2 ± 212.2	864.6 ± 29.1
IV	Endocrine, nutritional and metabolic diseases	2922.6 ± 79.6	6779.2 ± 258.9	1805.7 ± 42.1	2105.8 ± 67.6	4618.9 ± 213.7	484.4 ± 21.8
VI	Nervous system diseases	3561.8 ± 87.9	3036.6 ± 173.3	2974.3 ± 54.0	2409.5 ± 72.3	2120.8 ± 144.8	2082.8 ± 45.2
VII	Diseases of the eye and adnexa	9283.6 ± 141.9	9229.8 ± 302.1	9273 ± 95.4	7697.8 ± 129.2	6784.7 ± 259.0	4801.9 ± 68.7
VIII	Diseases of the ear and mastoid process	4481.2 ± 98.6	2709.7 ± 163.7	2191.4 ± 46.4	4367.0 ± 97.3	1659.4 ± 128.1	1607.8 ± 39.7
IX	Diseases of the circulatory system	592.9 ± 35.8	2783.4 ± 165.9	9067.1 ± 94.4	423.2 ± 30.3	1424.6 ± 118.7	2756.2 ± 52.0
X	Diseases of the respiratory system	55411.1 ± 346.6	14954 ± 384.6	11031.3 ± 104.1	45705.5 ± 314.8	13875.2 ± 370.4	9727.5 ± 97.7
XI	Diseases of the digestive system	13374.4 ± 170.3	17159.6 ± 412.0	8446.5 ± 91.2	12539.7 ± 164.9	12127.11 ± 346.3	5854.4 ± 75.8
XII	Diseases of the skin and subcutaneous tissue	13398.7 ± 170.4	6610.8 ± 255.7	6643.2 ± 80.8	11679.2 ± 159.1	5484.2 ± 232.9	5586.3 ± 74.1
XIII	Diseases of the musculoskeletal system	1964.2 ± 65.3	6335.9 ± 250.3	3680.2 ± 60.1	1596.5 ± 58.8	5230.7 ± 227.4	3036.9 ± 54.6
XIV	Diseases of the genitourinary system	1531.3 ± 57.6	5073.9 ± 224.0	5096.1 ± 70.7	1065.0 ± 48.0	3984.5 ± 198.5	4193.3 ± 64.2
XV	Pregnancy, delivery, postpartum period	-	160 ± 59.8	11431.5 ± 106.0	-	-	6111.8 ± 77.5
XVI	Certain perinatal conditions	6876.3 ±122.1	-	-	6876.3 ± 122.1	-	-
XVII	Congenital abnormalities	2643.7 ± 75.7	233.8 ± 48.0	99.5 ± 9.9	1130.1 ± 49.5	171.1 ± 41.1	23.6 ± 4.8
XIX	Injuries and intoxications	4354.3 ± 97.2	4371.1 ± 207.9	4029.8 ± 62.9	4012.2 ± 93.3	3297.0 ± 180.6	3384.7 ± 57.7

But at the same time the incidence of the genitourinary system and the number of nervous disorders increase, and the number of neoplasms increases. Men from Aktau had a similar situation. Among adolescents, metabolic and endocrine gland disorders were observed.

An assessment of the incidence of newly diagnosed diseases in the age group of children (Aktau) showed that, in comparison with the general distribution, the number of congenital anomalies decreased by approximately 1.5 times for the surveyed period. The high morbidity rate among children living in Aktau, compared with adolescents and adults, is indicative of the unfavorable environmental situation in the region, since children’s health is the main indicator of unfavorable environmental effect on health.

**Fig. 1:**
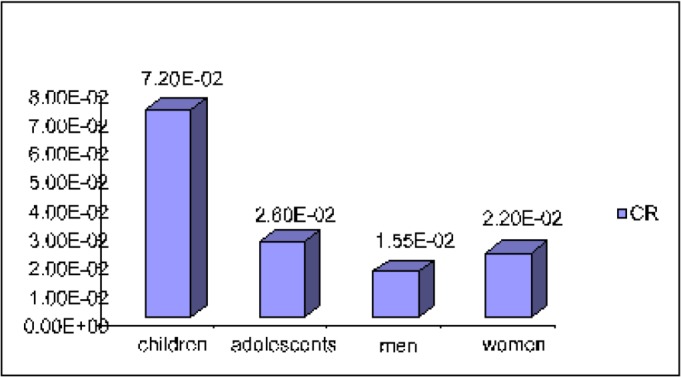
Individual carcinogenic risk for the population of Aktau with regard to age

**Fig. 2:**
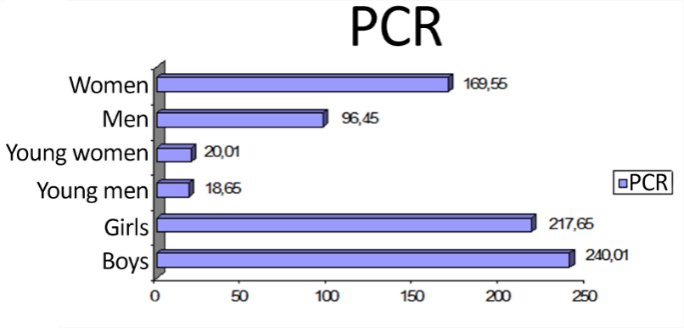
Population carcinogenic risks for the population of Aktau by gender and age

Analysis of the evaluation of individual and population carcinogenic risk (Figs. 1, 2), with regard to gender and age, showed that the child population (aged 0–6) of Aktau was at the highest risk of cancer. In comparison between girls and women (Fig. 2), the former have a higher carcinogenic risk.

Studies of non-carcinogenic effects produced by air pollution in the residential districts of Aktau showed that the highest non-carcinogenic risk under the effect of airborne chemical substances was found among the child population (aged 0–6), as shown by the integrated indexes of risk assessments – HQ, HI (Table 3)

**Table 3:** The nature of non-carcinogenic risk to the health of children (aged 0–6) in Aktau

***Substance***	***Dose, mg/kg***	***RfC, mg/kg***	***HQ***	***Organ***
Sulfur dioxide	0.026	0.66	0.111	respiratory system
Hydrogen sulfide	0.0007	0.002	1.0	respiratory system
Carbon monoxide	0.416	3.0	0.389	blood, cardiovascular system, development, CNS
Nitrogen oxide	0.346	0.47	2.059	respiratory system, blood (formation of MetHb)
Ammonia	0.47	0.1	1.313	respiratory system
Common hydrocarbons	0.939	0.071	37.023	eyes, respiratory system, liver, kidneys, CNS
Aggregate risk		HI total	**41.895**	
		HI development	0.389	
		HI kidneys	37.023	
		HI blood, cardiovascular system	2.448	
		HI respiratory system	41.506	
		HI CNS	37.412	
		HI liver	37.023	

In adolescents, a high risk of diseases of the respiratory system, kidney and liver, and the nervous system was found (Table 4). However, the level of risk to the health of adolescents was 2.5–3 times lower than that of children. For instance, the HQ for common hydrocarbons was 13.227, i.e. they were the only chemical substance with an HQ > 1. The general HI in this group was 14.971. In terms of diseases of critical organs and systems, the highest HI is of respiratory organ diseases (HI=14.832), followed by disorders of the central nervous system (HI=13.366), and kidneys and liver diseases (HI=13.227).

**Table 4:** The nature of non-carcinogenic risk to the health of adolescents (aged 14–18) in Aktau

***Substance***	***Dose, mg/kg***	***RfC, mg/kg***	***HQ***	***Organ***
Sulfur dioxide	0.026	0.66	0.039	respiratory system
Hydrogen sulfide	0.0007	0.002	0.36	respiratory system
Carbon monoxide	0.416	3.0	0.139	blood, cardiovascular system, development, CNS
Nitrogen oxide	0.346	0.47	0.736	respiratory system, blood (formation of MetHb)
Ammonia	0.047	0.1	0.47	respiratory system
Common hydrocarbons	0.939	0.071	13.227	eyes, respiratory system, liver, kidneys, CNS
Aggregate risk		HI total	**14.971**	
		HI development	0.139	
		HI kidneys	13.227	
		HI blood, cardiovascular system	0.875	
		HI respiratory system	14.832	
		HI CNS	13.366	
		HI liver	13.227	

The assessment of the population risk in Aktau by age groups (Table 5) found that the female population was exposed to the highest non-carcinogenic risk to health (Σ = 523.4), followed by adult men (Σ = 409.11), and adolescents (Σ = 207.33). With that, it is worth noting that the highest risk to public health comes from carbon monoxide and nitric oxide in the air.

**Table 5:** Non-carcinogenic population risk to the residents of Aktau

***Variable***	***Women***	***Men***	***Adolescents***
Sulfur dioxide	16.18	9.96	6.45
Hydrogen sulfide	0.44	0.33	0.17
Carbon monoxide	262.53	205.58	103.21
Nitrogen oxide	217.58	170.22	85.84
Ammonia	26.67	23.02	11.66
Σ	523.4	409.11	207.33

Obtained data on the LADD of hazardous substances in the air (at a consistent level of air pollution) indicate that the maximum daily concentrations throughout the entire life accrue to children aged 0–6, followed by adolescents, and adults (Table 6).

**Table 6:** LADD (lifetime average daily dose) of non-carcinogenic effects, mg/(kg*day)

***Variable***	***Children***	***Adolescents***	***Adults***
Sulfur dioxide	0.471	0.413	0.264
Hydrogen sulfide	0.013	0.011	0.007
Carbon monoxide	7.51	6.56	4.21
Nitrogen oxide	6.23	5.46	3.49
Ammonia	0.844	0.738	0.473
Common hydrocarbons	16.91	14.79	9.46
Volatile organic compounds	2.11	1.83	1.17
Σ	34.088	29.802	19.074

Obtained data on the lifetime average daily dose of hazardous substances in the air (at a consistent level of air pollution) are also a crucial indicator of the effect of adverse environmental conditions on the health of both children and adults.

## Discussion

Obtained results shows that the air pollution in studied urbanized areas affects the morbidity rate, its structure and prevalence. A high morbidity rate among children living in Aktau was discovered. The evaluation of the age- and gender-related features of the morbidity and its prevalence showed that boys fell ill 1.5 times more frequently than girls did; no significant differences were found in adolescents; in terms of adults, women fell ill 1.5–2 times more frequently than men did.

Numerous studies have confirmed the effect of unfavorable environmental factors, including atmospheric pollution, on public health (14, 20). With that, children are the first to experience this effect through increased general morbidity rates, respiratory and digestive diseases, and genitourinary system disorders (21). The heath indexes in city children, which were assessed in relation to unfavorable ecology, were worse than the ones in urban children (22). Deterioration of somatometric indexes and disharmonic development of adolescents living in a polluted environment was discovered (23, 24).

It is pertinent to point out that the poor and ethnic minorities are disproportionately exposed to ambient air pollutants, which are related to cardiovascular and respiratory disease, adverse perinatal outcomes, premature mortality etc. (25). Previously, scientists have determined the role of such sociopolitical factors as power, status, and alienation as strong determinants of people’s perception and acceptance of environmental risks (26).

The methodology for assessing the risk of environmental factors to human health is a novel interdisciplinary scientific area that is being actively developed. The results of assessment of individual and population non-carcinogenic risk for the Aktau population showed that the individual risk of cancer among all age and sex groups was very high. In both cases (individual and population risk), city children (aged 0–6) were the most vulnerable group. In our study, we did not study the factors of a specific occupational hazard. The system for assessing health risks enables using available monitoring data on environmental factors and public health to obtain both qualitative and quantitative characteristics of the environmental effect on public health before the onset of the consequences of such an effect. Furthermore, health risk assessment enables predicting the result and use this prediction to take managerial decisions to minimize the damage dealt by unfavorable environment to public health. Information on the morbidity rate allow optimizing programs aimed at preserving the health of population and improving the effectiveness of the medical social services.

Several manmade pollution areas of different origin have formed in the studied cities and regions. The studies found increased ground, water, and biological environment levels of chrome, boron, nickel, and lead near chrome industry facilities, of sulfides and vanadium near oil and gas mining and processing facilities (27–29). The strong toxic and specific action (30) of chrome and its compounds have a sensitizing and immunotoxic effect, which facilitates the growth of the rate of allergic diseases (31, 32) and malignancies (33).

The conducted research has shown that contamination of territories by chemical substances negatively influences the level of morbidity of the population. We believe that it is necessary to study in more detail the situation with pollution in this area and develop programs for improving the health of the population. Pollution of the external environment, and as a consequence, the increase in the incidence among residents of the region of pollution can undermine national development strategies and therefore it is so important to develop health programs and monitor the health of the population (34).

Recent studies also show the importance of a person’s lifestyle on the development of diseases. Sedentary lifestyle can be an additional factor in the development of cardiovascular diseases and type 2 diabetes (35). Therefore, in addition to such obvious environmental factors as the state of the external environment, the presence of pollutants, etc. It is necessary to take into account the way of life. Moreover, the entire environmental pathway from driving forces to health impact needs to be considered when designing interventions to improve the environment and health of children (36).

## Conclusion

The results show the need for a detailed study of the ecological situation and its impact on the health of people.

The system for assessing environmental risks is based on determining the health status of the population in order to predict coexisting diseases.

## Ethical considerations

Ethical issues (Including plagiarism, informed consent, misconduct, data fabrication and/or falsification, double publication and/or submission, redundancy, etc.) have been completely observed by the authors
